# Sleep disorders reveal distress among children and adolescents during the Covid-19 first wave: results of a large web-based Italian survey

**DOI:** 10.1186/s13052-021-01083-8

**Published:** 2021-06-04

**Authors:** Arianna Dondi, Anna Fetta, Jacopo Lenzi, Francesca Morigi, Egidio Candela, Alessandro Rocca, Duccio Maria Cordelli, Marcello Lanari

**Affiliations:** 1 Pediatric Emergency Unit, IRCCS Azienda Ospedaliero-Universitaria di Bologna, University of Bologna, Bologna, Italy; 2 IRCCS Istituto delle Scienze Neurologiche di Bologna, UOC Neuropsichiatria dell’età Pediatrica, Bologna, Italy; 3 Department of Medical and Surgical Sciences (DIMEC), S. Orsola Hospital, University of Bologna, Bologna, Italy; 4 Department of Biomedical and Neuromotor Sciences, Alma Mater Studiorum - University of Bologna, Bologna, Italy; 5 Specialty School of Pediatrics, Alma Mater Studiorum, University of Bologna, Bologna, Italy

**Keywords:** Coronavirus, Sleep, Mood changes, Child mental health, Lockdown, SDSC, Distance learning

## Abstract

**Background:**

Measures to contain the Covid-19 pandemic led to significant lifestyle changes for children and adolescents mainly related to the closure of schools and recreational activities, reduced social interaction, and increased family concerns.

**Methods:**

A cross-sectional online survey of 78 questions investigating social determinants of health, mood changes, symptoms of anxiety, increase in sleep disorders and unusual repetitive movements was offered to parents living in Italy with children ≤18 years; including families of children with disabilities, autism spectrum disorders, chronic diseases, and specific learning disabilities. The survey was conducted on the Qualtrics platform 6 months after the beginning of the pandemic and distributed in hospitals and paediatricians’ waiting rooms as well as through social networks. The primary outcomes were the increase in sleep disorders among children and adolescents. Possible risk factors were investigated through multivariable regression.

**Results:**

Six thousand two hundred ten volunteer parents responded to the questions concerning mood changes, sleep disorders and unusual repetitive movements, and were included in the present study. The majority were female (91.8%) and Italian (97.0%). 72.7% answered that their children had become more nervous, worried, or sad (80.2% in children with learning disabilities); 77.6% reported feelings of loneliness and 69.3% more difficulties in children falling asleep, 30.2% in staying asleep, and 18.7% an increase in nightmares and/or sleep terrors. Statistical analysis identified socioeconomic status, parent’s job loss, food insecurity, family attitude toward the pandemic, and children’s mood swing, feelings of loneliness, or missing outdoor activities, as major risk factors for sleep disorders.

**Conclusion:**

The first Covid-19 lockdown impacted children’s and adolescents’ health through an increase in sleep disorders. In the following phases of the pandemic, this evidence may be useful to investigate and treat these disorders as well as make decisions about containment health policies concerning this age group.

**Supplementary Information:**

The online version contains supplementary material available at 10.1186/s13052-021-01083-8.

## Background

The novel Coronavirus disease 2019 (COVID-19) has quickly spread across the globe since December 2019 and drastically changed daily life for billions of people. This pandemic and its associated societal response is thought to have wide-ranging impacts on youth development and child mental health [1]. Indeed, being forced to stay at home, reduce social interaction, minimize outings, boredom, and in parallel manage the attendant health risks, can have a major impact on psychological distress [2]. Quarantined people are significantly more likely to report exhaustion [3], depression [4], stress [5], low mood, irritability, insomnia, migraine [6] and post-traumatic stress symptoms [7]. A previous survey-based study comparing post-traumatic stress symptoms in parents and children quarantined (most of them because of H1N1 and SARS infection) with those not quarantined found that the mean post-traumatic stress scores were four times higher in the first group [8]. Sleep disorders are common in the paediatric age and their prevalence is estimated to be around 25% [9], with significant worsening in stressful situations [10]. There are few systematic studies of the impact of the current pandemic on children. Among these, some have found frequent alterations in sleep routines, increased total sleep duration, and negative impact on sleep quality [11, 12]. Youth with pre-existing neurodevelopmental conditions (including autism spectrum disorder, ASD) may also be particularly vulnerable to worsening of sleep disorders due to restrictive measures [13]. Sleep is an important factor that influences the cognitive, behavioural, and functional development of children [14–16]. It is therefore important to detect disturbances in the sleep-wake rhythm at an early stage in order to avoid short- and long-term consequences.

With this survey, we aimed to evaluate the effects of COVID-19 quarantine on sleep quality as an indicator of psychological well-being among children and adolescents living in Italy. Secondly, we aimed to identify potential familial, socioeconomic, and personal risk factors for their occurrence. Subpopulations of patients with learning disabilities, ASD, other disabilities, chronic conditions, and multiple conditions were also considered to specifically assess the impact of the pandemic on them.

## Methods

### Study design and survey development

An online cross-sectional survey of 78 questions was offered to a population made up of families living in Italy with children up to 18 years old. The questions were designed on the basis of a review of the scientific literature relating to the topic and extensive discussion between the authors.

The aim of the survey was to analyse some of the demographic variables of families and social determinants of health (housing, level of parental education, employment, hunger, stress) [17] among families with children. Briefly, we investigated the composition of the family, the characteristics of the dwelling, parental level of education and job characteristics (type of employment, working time commitment and changes in working conditions during the pandemic), changes in food security before and after the beginning of the pandemic through the Hunger Vital Sign (HVS) 2-item test [18], and modifications in eating habits and weight. Moreover, we inquired children’s sleep disturbances, the onset or worsening of unusual repetitive movements, mood changes, anxiety, and the relationship with school activities and online distance learning. The complete questionnaire is available in Additional file 1-Table A1.

For the purpose of the present study, we analysed the questions focusing on sleep quality and possible risk factors for its deterioration. In detail, the emergence or worsening of disorders of initiating sleep, maintaining sleep, and nocturnal awakenings after the pandemic outbreak (“Did you ever notice that at least one of your children having more difficulty falling asleep during this COVID-19 pandemic?”; “Does at least one of your children awake more than twice per night?”; “Did it ever happen in the past before the COVID-19 pandemic?”; “Did you ever notice that one or more of your children started talking and screaming anguished without waking up during the COVID-19 pandemic?”) were investigated by including some items of the Sleep Disturbance Scale for Children (SDSC) [19]. Moreover, we explored the presence of unusual repetitive movements (“Did you notice the appearance or worsening of unusual, repetitive movements (tics) in at least one of your children?”), mood changes, symptoms of anxiety and the psychophysical well-being of families, the relationship of families and students with school activities and their opinion about online distance learning and future school challenges.

These items were also analysed in a specific subclass of people made up of families with children affected by specific learning disabilities (impairment in reading/written expression/mathematics), ASD, other disabilities, chronic diseases and multiple conditions.

### Survey conduction and distribution

The online survey was conducted on the research platform managed by Qualtrics. The Qualtrics model allows researchers to develop surveys using the Qualtrics software and can produce online reliable data similarly to traditional telephone and in-person methods [20].

The survey was open from September 1 until October 15, 2020, but most of the responses (93.3%) were recorded between September 20 and October 4, 2020. It was distributed through a link and a QR code disseminated through posters affixed in our hospital and in the waiting rooms of the paediatricians’ offices as well as through social networks with a snowball sampling technique. The questionnaire was filled out anonymously by parents, spontaneously and with prior online informed consent. The participants were given no reward or incentive.

### Statistical analysis

Summary statistics were presented as frequencies and percentages. Multivariable logistic regression analysis was used to investigate which sociodemographic characteristics were associated with changes in sleep disorders and unusual repetitive movements after the pandemic. Due to the presence of missing covariate data, multiple imputation by chained equations was used to replace missing values with multiple sets of simulated values to complete the data (*m* = 20). Regression estimates from the multiple imputed sets were then combined into one overall estimate with an associated variance that incorporated the within- and between-imputation variability [21]. Covariates that were not significantly associated with the outcome with a significance level of *P* ≤ 0.15 at bivariate analyses were not included in multivariable regression [22, 23]. Due to the descriptive nature of the analysis, we did not perform any further automated selection of the variables to be included in the final multivariable models. No multi-collinearity issues were found. All data were analysed using Stata version 15 (StataCorp. 2017. Stata Statistical Software: Release 15. College Station, TX: StataCorp LP). The significance level was set at 5%; odds ratios from multivariable logistic regression were evaluated using the 2-sided Wald test.

### Ethics

The present study was approved by the Ethics Committee of the University Hospital of Bologna (Italy) (Institutional Review Board approval number 762/2020/Oss/AOUBo).

## Results

The survey was filled in by 7958 parents, but 6210 (78.0%) fully completed the questions concerning sleep disorders and unusual repetitive movements. The 1748 (22%) subjects excluded from the analysis had younger offspring, higher levels of unemployment, lower educational attainment, and were more pessimistic about their economic status after the pandemic (additional file 1- Table A2).

The socioeconomic characteristics of the study sample are summarized in Table 1, as well as parents’ experience and attitudes toward the COVID-19 pandemic. The majority of the respondents were female (91.8%), Italian (97.0%) and living in Northern Italy (89.1%). Either parent had a university degree and both parents were employed in 66.8 and 85.0% of the families, respectively. Learning disabilities were present in 4.3% of the respondents’ children, other disabilities in 1.5%, chronic conditions in 1.2%, ASD in 0.8%, and multiple conditions in 0.8%. Household economy was described as either “well-off” or “overall satisfactory” by 93.3% of the respondents. In 4.4% of the cases, either parent lost their job since the beginning of the pandemic. Almost 60% considered their means more difficult or at risk after the pandemic. Regarding their attitude during the lockdown, parents thought that their children had mainly missed meeting friends and outdoor activities, and reported that they had become more nervous, worried, or sad, and that they had had feelings of loneliness in 72.7 and 67.6% of cases, respectively.

**Table 1:** Parent’s sociodemographic characteristics and experience/attitudes towards the Coronavirus pandemic

Characteristic	***n = 6210***	%
Age, y	(no missing)	
≤30	307	4.9
31–35	1007	16.2
36–40	1795	28.9
41–45	1654	26.6
46–50	999	16.1
> 50	448	7.2
Sex	(no missing)	
Male	509	8.2
Female	5701	91.8
Country of origin	(30 missing)	
Italy	5995	97.0
Outside Italy ^(a)^	185	3.0
Area of residence ^(b)^	(12 missing)	
Northern Italy	5520	89.1
Central Italy	379	6.1
Southern Italy	299	4.8
Educational attainment of the parents	(17 missing)	
Both secondary school	2053	33.2
Secondary school & graduate school	2110	34.1
Both graduate school	2030	32.8
Working condition of the parents	(32 missing)	
Both unemployed	39	0.6
One unemployed	885	14.3
Both employed	5254	85.0
Job type	(no missing)	
Clerk	3036	48.9
Retired	805	13.0
Homemaker	744	12.0
Laborer	320	5.2
Freelancer	309	5.0
Health-care worker	146	2.4
Dealer	141	2.3
Other ^(c)^	709	11.4
Economic status	(3 missing)	
Well-off	2454	39.5
Somewhat difficult but overall satisfactory	3340	53.8
Quite difficult	384	6.2
Often unsustainable	29	0.5
Number of children in the family	(no missing)	
1	2639	42.5
2	2967	47.8
3	460	7.4
> 3	144	2.3
Age of the youngest or only child, y	(no missing)	
≤2	1980	31.9
3–5	1528	24.6
6–10	1561	25.1
11–14	706	11.4
> 14	435	7.0
Children with disorders or disabilities	(210 missing)	
No	5485	91.4
Learning disabilities	257	4.3
Other disabilities	90	1.5
Chronic conditions	70	1.2
Autism spectrum disorders	49	0.8
Multiple conditions	49	0.8
A member of the family got COVID-19	(1 missing)	
No	5395	86.9
Yes	437	7.0
Yes, hospitalized	203	3.3
Yes, passed away	174	2.8
Economic status after the outbreak	(no missing)	
Improved	168	2.7
Unchanged	3428	55.2
Slightly worsened	2303	37.1
Worsened	270	4.3
Become critical	41	0.7
Either parent has lost their job	(no missing)	
No	5938	95.6
Yes	272	4.4
How the parent sees her/his means after the pandemic	(1 missing)	
Better	142	2.3
Unchanged	2512	40.5
More difficult	3125	50.3
Much more difficult	359	5.8
Seriously at risk	71	1.1
Increased worry about running out of food	(3 missing)	
No	5563	89.6
Yes	644	10.4
Running out of food more often	(2 missing)	
No	6060	97.6
Yes	148	2.4
Changes in children’s food intake	(6 missing)	
No	3759	60.6
More food	1642	26.5
Less food	803	12.9
What have your children missed more? ^(d)^	(no missing)	
Going to school	1812	29.2
Outdoor activities	2845	45.8
Meeting friends	4878	78.6
Meeting relatives	2491	40.1
Playing sports	2497	40.2
Any mood swing in your children?	(5 missing)	
No	1529	24.6
Yes, more nervous, troubled, or sad	4509	72.7
Yes, their mood has improved	167	2.7
Did your children have feelings of loneliness?	(18 missing)	
No	2007	32.4
Yes, not putting it into words	1955	31.6
Yes, putting it into words	2230	36.0

^(a)^135 from Europe, 30 from the Americas, 11 from Africa and 8 from Asia (1 missing)

^(b)^ Northern Italy: Piedmont, Aosta Valley, Lombardy, Liguria, Trentino-South Tyrol, Veneto, Friuli-Venezia Giulia, Emilia-Romagna; Central Italy: Tuscany, Umbria, Marche, Lazio; Southern Italy: Abruzzo, Molise, Campania, Apulia, Basilicata, Calabria, Sicily, Sardinia

^(c)^ Including teachers, students, educators, farmers, police, unemployed, and unable to work

^(d)^Multi-select question (the sum of percentages exceeds 100)

**Fig. 1:**
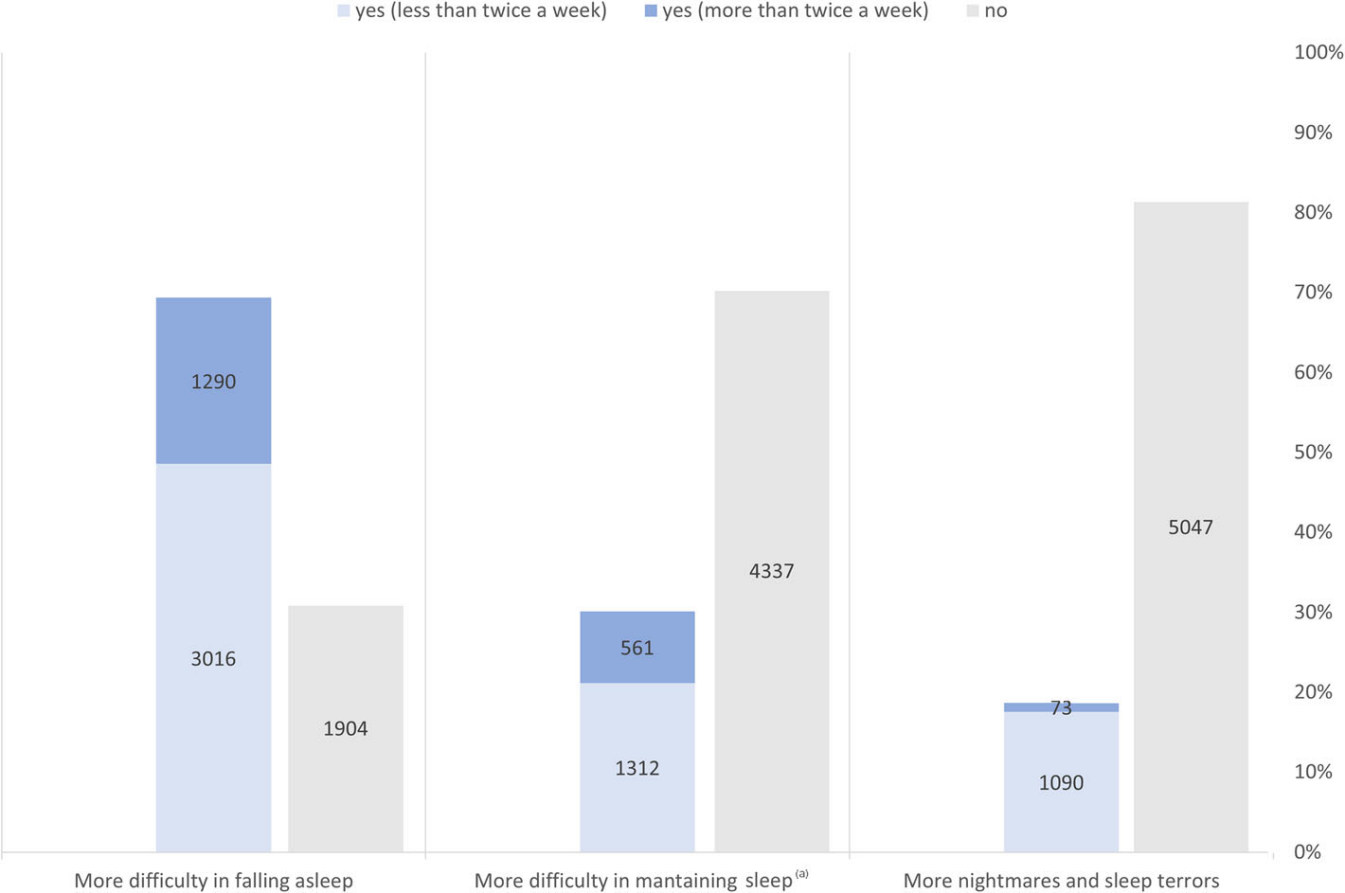
Increase in children’s sleep disorders after COVID-19 outbreak. The graph shows parents’ answers to the three reported questions. **a** More difficulty in maintaining sleep is defined as waking more than twice during the night

Changes in sleep disorders after the Coronavirus pandemic are illustrated in Fig. 1. In 4306 (69.3%) families, children had more difficulties falling asleep; the frequency of these episodes was more than twice a week in 1290 (30.0%) cases. In 1873 (30.2%) families, the children had more difficulties staying asleep; the frequency of these episodes was more than twice a week in 561 (30.0%) cases. An increased number of nightmares and/or sleep terrors was reported in 1163 (18.7%) families; the frequency of these episodes was more than twice a week in 73 (6.3%) cases.

Table 2 reports the multivariable logistic regression analysis for increased sleep disorders in children after the beginning of the pandemic. The analysis shows that household economic concerns, household food insecurity, and parents’ perception of increased difficulty in the family means after the pandemic were significantly associated with children’s sleep disorders. Modification in children’s food intake (both more and less food), mood changes (children having feelings of loneliness, sadness, worry, or nervousness) or missing outdoor activities were related to increased sleep disturbances. All these characteristics were linked both to difficulty in falling asleep and staying asleep during the night. Job loss by at least one of the parents and the presence of “chronic diseases” were found to be closely connected to difficulties in staying asleep, while difficulty in falling asleep was greater in those affected by “multiple conditions”. Similarly, children with mood swings, feelings of loneliness, diet changes, and family economic problems were significantly more likely to present nightmares or night terrors.

**Table 2:** Multivariable logistic regression analysis of increased sleep disorders in children after COVID-19 outbreak. Results are presented as odds ratios (standard errors)

Characteristic	Difficulty falling asleep	Difficulty staying asleep	Nightmares and terrors
Female sex (ref: male)	1.09 (0.12)	1.28 (0.16)*	·
Economic status (ref: well-off)			
Somewhat difficult but overall satisfactory	1.17 (0.08)*	1.14 (0.08)	1.09 (0.08)
Difficult/unsustainable	1.38 (0.22)*	1.38 (0.19)*	1.29 (0.19)
Age of the youngest or only child, y (ref: ≤2)			
3–5	·	0.72 (0.06)**	0.66 (0.06)**
6–10	·	0.54 (0.05)**	0.47 (0.04)**
11–14	·	0.45 (0.05)**	0.24 (0.04)**
> 14	·	0.59 (0.08)**	0.17 (0.04)**
Children with disorders or disabilities (ref: no)			
Learning disabilities	1.06 (0.17)	1.32 (0.20)	·
Other disabilities	1.01 (0.26)	1.51 (0.37)	·
Chronic conditions	1.52 (0.45)	1.83 (0.53)*	·
Autism spectrum disorders	1.05 (0.38)	1.42 (0.45)	·
Multiple conditions	2.60 (1.21)*	1.77 (0.57)	·
Unspecified	1.04 (0.18)	1.00 (0.17)	·
Job type (ref: health-care worker)			
Clerk	1.18 (0.24)	1.52 (0.32)*	·
Retired	1.21 (0.26)	1.37 (0.31)	·
Homemaker	1.04 (0.22)	1.41 (0.32)	·
Laborer	0.76 (0.18)	1.23 (0.30)	·
Freelancer	1.15 (0.28)	1.40 (0.34)	·
Dealer	1.37 (0.39)	1.15 (0.34)	·
Other	0.93 (0.20)	1.23 (0.28)	·
Either parent has lost their job (ref: no)	1.25 (0.22)	1.63 (0.24)**	1.48 (0.22)*
How the parent sees her/his means after the pandemic (ref: better/unchanged)			
More difficult	1.28 (0.08)**	1.40 (0.10)**	1.14 (0.09)
Much more difficult/seriously at risk	1.43 (0.21)*	1.60 (0.21)**	1.13 (0.16)
Increased worry about running out of food (ref: no)	1.06 (0.12)	1.09 (0.11)	1.08 (0.12)
Running out of food more often (ref: no)	2.21 (0.66)**	2.16 (0.43)**	1.31 (0.27)
Changes in children’s food intake (ref: no)			
More food	1.80 (0.14)**	1.83 (0.13)**	1.56 (0.12)**
Less food	2.02 (0.21)**	2.16 (0.19)**	1.62 (0.16)**
Children have missed going to school (ref: no)	·	1.13 (0.08)	0.88 (0.07)
Children have missed outdoor activities (ref: no)	1.43 (0.09)**	1.16 (0.07)*	1.26 (0.09)**
Children have missed meeting friends (ref: no)	1.14 (0.09)	0.92 (0.07)	1.08 (0.08)
Children have missed playing sports (ref: no)	0.89 (0.06)	·	0.92 (0.07)
Any mood swing in your children? (ref: no)			
Yes, more nervous, troubled, or sad	3.16 (0.22)**	4.85 (0.52)**	2.11 (0.23)**
Yes, their mood has improved	0.87 (0.15)	0.45 (0.18)*	1.23 (0.35)
Did your children have feelings of loneliness? (ref: no)			
Yes, not putting it into words	1.85 (0.14)**	1.83 (0.16)**	2.10 (0.21)**
Yes, putting it into words	1.97 (0.15)**	2.11 (0.18)**	2.05 (0.20)**
Constant	0.28 (0.07)**	0.03 (0.01)**	0.08 (0.01)**

Note: “·” denotes independent variables there were discarded in preliminary bivariate analysis (*P* > 0.15)

*Significant at the 5% level (*P* ≤ 0.05); **Significant at the 1% level (*P* ≤ 0.01)

Among the 730 (11.8%) families complaining of an increase in their children’s unusual repetitive movements after the outbreak, 514 (70.4%) reported new-onset, while 216 (29.6%) worsening of pre-existing symptoms. The logistic regression analysis revealed that the worsening of mood was associated both with an increase in pre-existing unusual repetitive movements (OR = 2.77, *P* < 0.001) and the occurrence of new ones (OR = 1.56, *P* = 0.002); the same applied to the occurrence of feelings of loneliness that could not be verbalized (worsening of unusual repetitive movements: OR = 1.89, *P* = 0.006; new ones: OR = 1.49, *P* = 0.003). Aggravation of the symptoms was greater in children with ASD (OR = 7.24, *P* < 0.001) and other disabilities (OR = 5.85, *P* < 0.001).

About distance learning, contingency tables showed that children with learning disabilities were more likely to experience sadness, nervousness, or trouble (*P* < 0.001) and, in parallel, had more difficulty in paying attention during distance-learning classes (*P* < 0.001). Additional file 2 reports complete analysis on children’s unusual repetitive movements (Table A3) and distance learning (Table A4).

## Discussion

This questionnaire-based study, involving 6210 parents of individuals aged 0–18, is, to date, the largest study about children and adolescents’ experience during the COVID-19 pandemic and the effects on their physical and mental health.

Similar to other countries, Italy experienced a total lockdown from March 11 to May 18, 2020. As expected in a period of isolation, uncertainty and general concern, feelings of loneliness, sadness, or trouble were present in most of children and adolescents.

One of the most striking finding in our study is the increase in sleep disorders relating to problems with falling asleep, maintaining sleep, and the presence of nightmares and/or sleep terrors.

As our questionnaire asked for the “age of the youngest child” and the questions focused on all children in the household, we were not able to make an exact breakdown by age group. Consequently, this result is hardly comparable to the few existing data in the literature: all but one of them concern pre-school children (aged 3–6). Moreover, they all have smaller samples and use different parameters. Some of them report a negative impact on sleep quality with alterations in sleep routines: a French study on 92 children (median age 29 months) highlighted an increased total SDSC score during the lockdown, with major difficulties in initiating and maintaining sleep, and an increased frequency of parasomnias [24]. An Italian study on 37 children aged 3–6, carried out during lockdown through repeated parental reports, found that after an initial phase of worsened sleep, there was a stabilization of its routine and quality [11]. Others found no effect of the lockdown: a study on 1619 Chinese children aged 4–6 years showed no difference in the Children’s Sleep Habit Questionnaire (CSHQ) scores compared to old surveyed in 2018 [25]. A smaller Italian retrospective study found no significant effect of the lockdown on SDSC score [26]. A larger (*n* = 1472) Canadian survey focusing on school children and youth (5–17 years) showed no differences in terms of sleep quality but reported increased total sleep duration, more evident in adolescents than in children [12].

Sleep disorders are relatively frequent in paediatric populations and are linked to behavioural and emotional problems both in children and adolescents [27–30]. Isolation imposed during the lockdown may compromise children’s ability to successfully regulate behaviour and emotions and consequently there is potential for sleep problems to emerge or worsen [31]. Our results confirm this hypothesis: we found a higher frequency of sleep disturbances among those children and adolescents in our population who appear to experience feelings of loneliness, sadness, worry, or nervousness. Sleep disorders were also found to be linked to the presence of other factors “unmasking” the distress in children and adolescents such as changes in food intake.

Social and psychosocial factors also appear to influence sleep disturbance. We found that the perception of family economic instability and job insecurity, even without a change in the employment situation during the pandemic, is a risk factor for problems related to the initiation and maintenance of sleep. This result can be directly traced to the known influence that anxiety, depression (especially maternal) and parental stress have on toddlers’ sleep [26, 32], and, on the other hand, in older children and adolescents, to a direct understanding of family experience, spells of unemployment and parental concern [3]. Before this pandemic, several authors had reported financial loss as a risk factor for psychological disorders and both anger and anxiety during and after quarantine [33–35]. It has also been suggested that sleep disorders in children manifest a socio-economic gradient, the causes of which, however, have not been identified [36]. It was thus not surprising that, in the case of parental job loss, also nightmares and terrors occurred more frequently, revealing a much deeper state of concern in the child/adolescent. As some authors report, the inability to make meaning of traumatic or unusual elements may lead to misleading or biased cognitive appraisal and emotional overreactions [37], that may contribute to the pathogenesis of sleep disturbance.

About a possible selection bias, we interestingly found that some socio-economic elements significantly associated with higher child distress (i.e. difficult economic status and/or worsened after the outbreak, parental job loss) were more frequent among excluded individuals whose parents did not provide information about sleep disturbances, so the prevalence of these conditions in the general population may be higher than what we estimated. However, we cannot exclude that respondents to the questionnaire possess cognitive, behavioural, and cultural traits that increase their levels of attention and anxiety towards their offspring’s health conditions, leading to an overestimation of all prevalence figures in our study sample.

Unexpectedly, children of healthcare workers (2.4% of our sample) directly involved in pandemic management and usually experiencing chronic stress and higher levels of depression and anxiety [38], did not show a higher rate of sleep disorders.

In our population, difficulty in falling asleep, nocturnal awakenings, and nightmares and/or sleep terrors were higher in children who were reported to miss outdoor activity. Outdoor activity is related to a better quality of sleep [39]; a recent study also demonstrated that sufficient time spent outdoors is associated with a decreased risk of inadequate sleep time in children. The association seems to be age- and gender-dependent, being stronger in 6–13 year-old males [40]. We could therefore hypothesize that the sleep of a sub-group of children more prone to physical activity (and therefore missing it more) can be affected even more negatively by lockdown and consequent reduced outdoor activity.

Moreover, the inability to go outside, remote learning, and the absence of in-person social interactions lead to a higher amount of time spent using technology, even during the pre-sleep period [13]. The reduced exposure to the sunlight and the prolonged exposure to screen blue and bright light may contribute to determine the sleep disorder by disrupting the physiological circadian rhythm [39–43].

About additional possible risk factors, Becker and Gregory have suggested that youth with pre-existing psychopathologies and neurodevelopmental conditions may be particularly vulnerable to disturbed sleep during this pandemic [13]. In our survey we investigated 6 groups: learning disabilities, ASD, other disabilities, chronic conditions, and multiple conditions. No differences between these groups have been found except for a higher rate of difficulty in staying asleep in the “chronic disease” group and of difficulty in falling asleep in the “multiple conditions” group. Unfortunately, since these two groups include patients with very different characteristics, no further speculation is possible.

Regarding unusual repetitive movements we interestingly observed an increased incidence among children with ASD and other disabilities. We therefore suggest that in these cases the “ unusual repetitive movements” may be stereotypies (rather than tics as suggested in the questionnaire), which are frequent in patients with ASD and severe cognitive impairment [44] and might be increased as an expression of anxiety and distress in these patients [45–48]. However, further specific studies with targeted questions are necessary to confirm this supposition.

Finally, an interesting fact to note, although not univocally explainable with our data, is the higher rate of feelings of loneliness, sadness, or trouble in the group of children with specific learning disorders. Their parents also reported a greater discomfort compared to children without other pathologies or disabilities in paying attention during distance-learning classes. They also expressed feelings of inadequacy in supporting their children’s distance learning and helping them manage their anxiety related to it. Unfortunately, the number of children with learning disabilities is too low to reach a conclusion. Further targeted studies could be useful to clarify this point, as a higher stress rate in engaging distance learning has already already been reported in case of specific learning disorders [49].

Our study has several limitations. The first possible bias is that, as this survey was promoted predominantly by social media, we cannot know the real number of people who received the invitation to participate in the survey and, consequently, the response rate. Moreover, such a study design could limit the ability to reach groups that do not have access to the Internet and thus exclude a population that might be particularly at risk of suffering from the pandemic aftermath. Finally, the inherent limitation of parent response-based questionnaires may not always coincide with the child’s perception and be influenced by numerous factors including parental stress itself.

## Conclusions

With this study, we investigated the impact of COVID-19 emergency measures on children and adolescents’ well-being, and the possible risk factors of distress. We found an increase in sleep disorders that significantly impact their quality of life. The following phases of the pandemic are again requiring severe restrictions, including distance learning and social contact limitations, to control the spread of the infection, and it is, therefore, essential to investigate and treat these disorders to prevent long-term physical, cognitive, and psychological effects.

We believe that our results can be of support in formulating public health measures impacting children and adolescents such as outdoor activity restrictions, considering the increased vulnerability of these developing individuals. We also call for concrete and direct measures to support families and children, with an emphasis on those with physical and mental frailty.

## Supplementary Information

**Additional file 1: Table A1.** Online questionnaire. **Table A2.** Percentage distribution of the characteristic of subjects included and excluded from the analyses.

**Additional file 2: Table A3.** Multivariable logistic regression analysis of the increase in children’s unusual repetitive movements after COVID-19 outbreak. **Table A4.** Percentage distribution of mood swings and information about distance learning, by child’s condition.

## Data Availability

All data and materials are available at Sant’Orsola University Hospital, Bologna in AD’s office.

## Abbreviations

COVID-19: Coronavirus Disease 2019
ASD: Autism Spectrum Disorder
SDSC: Sleep Disturbance Scale for Children
