# Supplementary material for: Sleep disorders reveal distress among children and adolescents during the Covid-19 first wave: results of a large web-based Italian survey
Source: Ital J Pediatr. 2021 Jun 4;47:130. doi: 10.1186/s13052-021-01083-8 (PMC8176278; doi:10.1186/s13052-021-01083-8)
Supplement: Supplementary file 1 — Additional file 1: Table A1. Online questionnaire. Table A2. Percentage distribution of the characteristic of subjects included and excluded from the analyses. [file 13052_2021_1083_MOESM1_ESM.docx]

**Additional File 1.**

**Table A1. Online questionnaire**

| **Questions** | **Answers** |
| --- | --- |
| **What is your age group?** | [__] 18-20 |
|  | [__] 21-25 |
|  | [__] 26-30 |
|  | [__] 31-35 |
|  | [__] 36-40 |
|  | [__] 41-45 |
|  | [__] 46-50 |
|  | [__] 51-59 |
|  | [__] 60 or more |
| **Gender** | [__] male |
|  | [__] female |
| Home country of the parent filling the questionnaire | [__] Italy |
|  | [__] not Italy |
| If not Italy, what country? | [_________________________________] |
| How long has the parent filling the questionnaire lived in Italy? | [__] less than 6 months |
|  | [__] more than 6 months but less than 2 years |
|  | [__] more than 2 years but less than 5 |
|  | [__] more than 5 years |
| Are you an Italian citizen? (parent filling the questionnaire) | [__] yes |
|  | [__] no |
| Home country of the other parent | [__] Italy |
|  | [__] not Italy |
|  | [__] single parent |
| If not Italy, what country? | [______________________] |
| Since when is the other parent living in Italy? | [__] less than 6 months |
|  | [__] more than 6 months but less than 2 years |
|  | [__] more than 2 years but less than 5 |
|  | [__] more than 5 years |
|  | [__] single parent |
| Does the other parent have Italian citizenship? | [__] yes |
|  | [__] no |
| In what Region do you live? | [_______________________] |
| What Province? | [_______________________] |
| Do parents live together? | [__] yes |
|  | [__] no |
|  | [__] single parent |
| **Number of children less than 3-year-old** | [__] none |
|  | [__] one |
|  | [__] two |
|  | [__] three |
|  | [__] more than three |
| **Number of children more than 3-year-old but less than 5-year-old** | [__] none |
|  | [__] one |
|  | [__] two |
|  | [__] three |
|  | [__] more than three |
| **Number of children more than 5-year-old but less than 10-year-old** | [__] none |
|  | [__] one |
|  | [__] two |
|  | [__] three |
|  | [__] more than three |
| **Number of children more than 10-year-old but less than 14-year-old** | [__] none |
|  | [__] one |
|  | [__] two |
|  | [__] three |
|  | [__] more than three |
| **Number of children more than 14-year-old but less than 10-year-old** | [__] none |
|  | [__] one |
|  | [__] two |
|  | [__] three |
|  | [__] more than three |
| Are you sharing home with other relatives? | [__] grandparents |
|  | [__] uncles |
|  | [__] cousins |
|  | [__] not sharing with other relatives |
| About your home: where are you living? | [__] my property |
|  | [__] I am renting |
|  | [__] with relatives, friends, or acquaintances |
|  | [__] hotel/pension at my expenses |
|  | [__] reception centre |
| How wide is your home (roughly)? | [__] less than 50 m² |
|  | [__] 51-70 m² |
|  | [__] 71-100 m² |
|  | [__] 101-150 m² |
|  | [__] more than 150 m² |
| How many bathrooms in your apartment? | [__] one |
|  | [__] two |
|  | [__] more than two |
| Do you have a garden (private or communal) or a terrace? | [__] yes |
|  | [__] no |
| What is your highest educational level? (Parent filling the questionnaire) | [__] none |
|  | [__] primary school (5th grade) |
|  | [__] middle school (8th grade) |
|  | [__] high school (12th-13th grade) |
|  | [__] university degree |
|  | [__] post-university degree |
| What is the highest educational level of the other parent? | [__] none |
|  | [__] primary school (5th grade) |
|  | [__] middle school (8th grade) |
|  | [__] high school (12th-13th grade) |
|  | [__] university degree |
|  | [__] post-university degree |
| About parent working conditions | [__] both unemployed |
|  | [__] one only is employed |
|  | [__] both are employed |
| **Which of the following best describes your type of job?** | [__] clerk |
|  | [__] worker |
|  | [__] dealer |
|  | [__] free lancer |
|  | [__] health care worker |
|  | [__] student |
|  | [__] not employed but looking for occupation |
|  | [__] not employed and NOT looking for occupation |
|  | [__] retiree |
|  | [__] unable to work |
|  | [__] other (specify) [___________________] |
| In this period, did at least one of the parents start with Smart Working? | [__] yes |
|  | [__] no |
| In this period, did at least one of the parents benefit from layoff? | [__] yes |
|  | [__] no |
| If working, how many hours per day? | [__] from 1 to 6 hours/day |
|  | [__] from 6 to 10 hours/day |
|  | [__] more than 10 hours/day |
| **How would you define your economic status?** | [__] well-off |
|  | [__] somewhat difficult but overall satisfactory |
|  | [__] quite difficult |
|  | [__] often unsustainable |
| **How did the overall economic situation of your family change during the COVID-19 pandemic?** | [__] improved |
|  | [__] remained the same |
|  | [__] slightly worsened |
|  | [__] worsened |
|  | [__] became critical |
|  | [__] no |
| **If you replied slightly worsened, worsened, or became critical: did one or more of your family lose his/her job due to the COVID-19 pandemic?** | [__] yes, the mother lost her job |
|  | [__] yes, the father lost his job |
|  | [__] yes, both parents lost their jobs |
| **How do you see the economic status of your family in the future because of the COVID-19?** | [__] better |
|  | [__] unchanged |
|  | [__] more difficult |
|  | [__] much more difficult |
|  | [__] seriously at risk |
| **Did one of your relatives, or somebody dear to you, get infected by COVID-19?** | [__] yes |
|  | [__] yes, and has been hospitalized |
|  | [__] yes, and died |
|  | [__] no |
| **Within the past 12 months before the pandemic (before March 2020) we worried whether our food would run out before we got money to buy more**. | [__] often |
|  | [__] sometimes |
|  | [__] never happened |
| **During this pandemic period (from March 2020 until today) we worried whether our food would run out before we got money to buy more** | [__] often |
|  | [__] sometimes |
|  | [__] never happened |
| **Within the past 12 months before the pandemic (before March 2020) the food we bought just did not last and we did not have money to get more.** | [__] often |
|  | [__] sometimes |
|  | [__] never happened |
| **During this pandemic period (from March 2020 until today) the food we bought just did not last and we did not have money to get more.** | [__] often |
|  | [__] sometimes |
|  | [__] never happened |
| **During this period when schools and all activities were closed, what do you think that your children missed more? (more than one answer is possible)** | [__] schooling |
|  | [__] outdoor activities |
|  | [__] meeting friends |
|  | [__] meeting relatives |
|  | [__] sports and other outdoor activities |
| **Did you notice any mood swing in one or more of your children during this COVID-19 pandemic?** | [__] yes, they are more nervous and restless |
|  | [__] yes, they look more sad |
|  | [__] yes, their mood has improved |
|  | [__] no, their mood has not changed |
| **Did you ever notice that at least one of your children having more difficulty falling asleep during this COVID-19 pandemic?** | [__] never |
|  | [__] occasionally (once or twice per month) |
|  | [__] sometimes (once or twice per week) |
|  | [__] often (3 to 5 times/week) |
|  | [__] always (every day) |
| **Does at least one of your children awake more than twice per night?** | [__] never |
|  | [__] occasionally (once or twice per month) |
|  | [__] sometimes (once or twice per week) |
|  | [__] often (3 to 5 times/week) |
|  | [__] always (every day) |
| **Did it ever happen in the past before the COVID-19 pandemic?** | [__] yes, in the same manner |
|  | [__] yes, but I have noticed the number of awakenings has increased and the sleep is more restless |
|  | [__] yes, but I have noticed a reduction in the number of awakenings |
| **Did you ever notice that one or more of your children started talking and screaming anguished without waking up during the COVID-19 pandemic?** | [__] never |
|  | [__] occasionally (once or twice per month) |
|  | [__] sometimes (once or twice per week) |
|  | [__] often (3 to 5 times/week) |
|  | [__] always (every day) |
| **Have you noticed any change in eating habits in at least one of your children during the COVID-19 pandemic?** | [__] yes |
|  | [__] no |
| **If yes, what was modified?** | [__] increased the amount of food |
|  | [__] reduced the amount of food |
| If the intake of calories was increased, what has been eaten particularly? (more than one answer possible) | [__] more snacks |
|  | [__] more carbonated drinks |
|  | [__] fruit juices |
|  | [__] other |
| Did you notice a change of body weight in at least one of your children? | [__] no, remained unchanged |
|  | [__] yes, weight has increased |
|  | [__] yes, weight has decreased |
| **Did you notice the appearance or worsening of unusual, repetitive movements (tics) in at least one of your children?** | [__] new appearance |
|  | [__] worsening |
|  | [__] not sure |
|  | [__] no |
| **Do you have schooling children who have taken advantage of distance learning through Internet?** | [__] yes |
|  | [__] no |
| If your answer is yes, what kind of digital media do you have at home for on-line distance learning? |  |
| (__) Computer | [__] none |
|  | [__] one |
|  | [__] two |
|  | [__] three |
|  | [__] more than three |
| (__) Tablet | [__] none |
|  | [__] one |
|  | [__] two |
|  | [__] three |
|  | [__] more than three |
| (__) Smartphone | [__] none |
|  | [__] one |
|  | [__] two |
|  | [__] three |
|  | [__] more than three |
| **Do you think that your digital skills are enough to support online distance learning of your children?** | [__] yes |
|  | [__] no |
| What has been the major obstacle for your children to follow the online distance learning? | [__] lack of adequate digital media |
|  | [__] more than one lesson at the same timing |
|  | [__] problems about Internet connectivity |
|  | [__] focusing on the topic and difficulty in following the lesson |
|  | [__] clash between parents working duties and need to support children in following their lessons |
|  | [__] other (none of the above) |
|  | [__] no problems with online distance learning |
| Have you been able to support your children with on-line distance learning? | [__] yes, often |
|  | [__] yes, sometimes |
|  | [__] never |
| As a parent, did you ever feel not up to the job to assist your children for their school commitments? | [__] yes, often |
|  | [__] yes, sometimes |
|  | [__] never |
| If your answer is yes, could you explain (more than one answer possible) | [__] could not provide adequate digital instruments |
|  | [__] not being able to use digital instruments |
|  | [__] not having an adequate educational level to provide support |
|  | [__] language barrier |
|  | [__] clash between personal working hours and children’s schooling time |
| **As a parent, did you ever feel inadequate to keep children anxiety under control?** | [__] yes |
|  | [__] no |
| If yes, why? (more than one answer possible) | [__] I was anxious too |
|  | [__] did not spend enough time with them |
|  | [__] I felt unfit |
|  | [__] I became impatient because they made me nervous |
|  | [__] uncertainties about the future |
|  | [__] fear of losing my job |
|  | [__] fear of the socio-economic consequences of the pandemic |
|  | [__] fear of getting infected |
|  | [__] fear of my dear getting infected |
| Did it happen more often to you to lose temper with your children in this period? | [__] yes |
|  | [__] no |
| If yes, did you more often resort to punishments, even corporal? | [__] yes |
|  | [__] no |
| Did you find yourself more often in disagreement with your partner on how to behave with your children? | [__] yes |
|  | [__] no |
| Did you fight more often with your partner? | [__] yes, with the partner |
|  | [__] yes, with my child/children |
|  | [__] yes, with both |
|  | [__] no |
| **Do you have children with disabilities, autism spectrum disorders, chronic diseases, specific learning disabilities (impairment in reading/written expression/mathematics)?** | [__] no |
|  | [__] yes, disabilities |
|  | [__] yes, autism spectrum disorders |
|  | [__] yes, chronic diseases |
|  | [__] yes, specific learning disabilities (impairment in reading/written expression/mathematics) |
| **Based on the type of disability: did they receive specific support for distance schooling?** | [__] yes |
|  | [__] no |
| Have you talked to the children about the return to school? | [__] yes |
|  | [__] no |
|  | [__] I have no school children |
| If so, what emotions emerged? (Multiple answers possible) | [__] fear |
|  | [__] uncertain |
|  | [__] confusion |
|  | [__] desire to return |
|  | [__] other |
| If you have school-age children (from primary school onwards), what do you see favourably for going back to school? (Multiple answers possible) | [__] I have no school-aged children |
|  | [__] mix of classroom attendance and online lessons |
|  | [__] only online lessons |
|  | [__] double shifts to avoid online lessons |
|  | [__] search for other innovative solutions including online lessons |
|  | [__] search for other innovative solutions excluding online lessons |
| Do you consider online teaching a useful tool to be used in the future to support personal teaching? | [__] yes  [__] no |
| If so, how could it be improved? (Multiple answers possible) | [__] train the children |
|  | [__] train teachers |
|  | [__] train parents |
|  | [__] infrastructure the digital network |
|  | [__] insert a government measure to allow the less well-off to have free resources (internet connection and/or devices) |
|  | [__] use cooperative learning methodologies to encourage the participation of children and young people |
| Do you think that during this health emergency the government has taken into consideration and managed the needs of children and young people? | [__] yes |
|  | [__] only partially |
|  | [__] no |
| What would you suggest as policies in favour of children and young people? (Multiple answers possible) | [__] nothing |
|  | [__] psychological support in school |
|  | [__] schools open to the community for full time, homework, and alternative activities |
|  | [__] greater involvement of parents in school decisions |
|  | [__] parents' participation in school activities open to the community |
|  | [__] greater involvement of children and young people in school decisions |
|  | [__] greater involvement of children and young people in school decisions |
|  | [__] structuring a model of participation of children and young people so that their requests reach the policymakers |
|  | [__] other |

Caption: The table shows the questionnaire in its complete form, as it was administered to the households. The questions used for the present study are in bold.

**Table A2. Percentage distribution of the characteristic of subjects included and excluded from the analyses.**

| **Characteristic** | **Complete information about sleep and tics** | |
| --- | --- | --- |
|  | **Yes (*n* = 6210)** | **No (*n* = 1748)** |
| Age, y |  |  |
| ≤30 | 4·9% | 8·5% |
| 31–35 | 16·2% | 17·5% |
| 36–40 | 28·9% | 25·9% |
| 41–45 | 26·6% | 23·2% |
| 46–50 | 16·1% | 13·9% |
| >50 | 7·2% | 11·0% |
| Sex |  |  |
| Male | 8·2% | 7·8% |
| Female | 91·8% | 92·2% |
| Country of origin |  |  |
| Italy | 97·0% | 95·0% |
| Outside Italy | 3·0% | 5·0% |
| Area of residence |  |  |
| Northern Italy | 89·1% | 84·6% |
| Central Italy | 6·1% | 6·4% |
| Southern Italy | 4·8% | 9·0% |
| Educational attainment of the parents |  |  |
| Both secondary school | 33·2% | 39·3% |
| Secondary school & graduate school | 34·1% | 30·0% |
| Both graduate school | 32·8% | 30·7% |
| Working condition of the parents |  |  |
| Both unemployed | 0·6% | 1·7% |
| One unemployed | 14·3% | 23·8% |
| Both employed | 85·0% | 74·5% |
| Job type |  |  |
| Clerk | 48·9% | 36·5% |
| Retired | 13·0% | 10·0% |
| Homemaker | 12·0% | 16·4% |
| Labourer | 5·2% | 8·3% |
| Freelancer | 5·0% | 8·5% |
| Health-care worker | 2·4% | 4·8% |
| Dealer | 2·3% | 2·8% |
| Other | 11·4% | 11·2% |
| Economic status |  |  |
| Well-off | 39·5% | 35·9% |
| Somewhat difficult but overall satisfactory | 53·8% | 52·6% |
| Quite difficult | 6·2% | 10·8% |
| Often unsustainable | 0·5% | 0·7% |
| Number of children in the family |  |  |
| 1 | 42·5% | 47·6% |
| 2 | 47·8% | 40·5% |
| 3 | 7·4% | 8·0% |
| >3 | 2·3% | 3·2% |
| Age of the youngest or only child, y |  |  |
| ≤2 | 31·9% | 42·3% |
| 3–5 | 24·6% | 19·3% |
| 6–10 | 25·1% | 20·5% |
| 11–14 | 11·4% | 10·1% |
| >14 | 7·0% | 7·8% |
| Children with disorders or disabilities |  |  |
| No | 91·4% | 89·4% |
| Learning disabilities | 4·3% | 4·8% |
| Other disabilities | 1·5% | 2·9% |
| Chronic conditions | 1·2% | 0·6% |
| Autism spectrum disorders | 0·8% | 0·3% |
| Multiple conditions | 0·8% | 1·9% |
| A member of the family got COVID-19 |  |  |
| No | 86·9% | 84·7% |
| Yes | 7·0% | 9·7% |
| Yes, hospitalized | 3·3% | 2·7% |
| Yes, died | 2·8% | 2·9% |
| Economic status after the outbreak |  |  |
| Improved | 2·7% | 1·9% |
| Unchanged | 55·2% | 49·9% |
| Slightly worsened | 37·1% | 39·7% |
| Worsened | 4·3% | 7·3% |
| Become critical | 0·7% | 1·2% |
| Either parent has lost their job |  |  |
| No | 95·6% | 93·9% |
| Yes | 4·4% | 6·1% |
| How the parent sees her/his means after the pandemic |  |  |
| Better | 2·3% | 2·3% |
| Unchanged | 40·5% | 33·3% |
| More difficult | 50·3% | 52·4% |
| Much more difficult | 5·8% | 10·0% |
| Seriously at risk | 1·1% | 2·0% |
| Increased worry about running out of food |  |  |
| No | 89·6% | 94·8% |
| Yes | 10·4% | 5·2% |
| Running out of food more often |  |  |
| No | 97·6% | 98·5% |
| Yes | 2·4% | 1·5% |
| Changes in children’s food intake |  |  |
| No | 60·6% | 56·9% |
| More food | 26·5% | 29·1% |
| Lees food | 12·9% | 14·0% |
| What have your children missed more?* |  |  |
| Going to school | 29·2% | 26·9% |
| Outdoor activities | 45·8% | 49·6% |
| Meeting friends | 78·6% | 69·7% |
| Meeting relatives | 40·1% | 38·2% |
| Playing sports | 40·2% | 36·5% |
| Any mood swing in your children? |  |  |
| No | 24·6% | 27·1% |
| Yes, more nervous, troubled, or sad | 72·7% | 71·8% |
| Yes, their mood has improved | 2·7% | 1·1% |
| Did your children have feelings of loneliness? |  |  |
| No | 32·4% | 34·0% |
| Yes, not putting it into words | 31·6% | 29·6% |
| Yes, putting it into words | 36·0% | 36·4% |
