# Supplementary material for: Sleep disorders reveal distress among children and adolescents during the Covid-19 first wave: results of a large web-based Italian survey
Source: Ital J Pediatr. 2021 Jun 4;47:130. doi: 10.1186/s13052-021-01083-8 (PMC8176278; doi:10.1186/s13052-021-01083-8)
Supplement: Supplementary file 2 — Additional file 2: Table A3. Multivariable logistic regression analysis of the increase in children’s unusual repetitive movements after COVID-19 outbreak. Table A4. Percentage distribution of mood swings and information about distance learning, by child’s condition. [file 13052_2021_1083_MOESM2_ESM.docx]

**Additional file 2.**

**Table A3. Multivariable logistic regression analysis of the increase in children’s unusual, repetitive movements after COVID-19 outbreak. Results are presented as odds ratios (OR) and standard errors (SE).**

| **Characteristic** | **New onset** | | **Worsening of symptoms** | |
| --- | --- | --- | --- | --- |
|  | **OR** | **SE** | **OR** | **SE** |
| Female sex (ref: male) | 1.23 | 0.24 | 2.02 | 0.75 |
| Economic status (ref: well-off) |  |  |  |  |
| Somewhat difficult but overall satisfactory | 0.99 | 0.10 | 1.01 | 0.17 |
| Difficult/unsustainable | 1.01 | 0.21 | 1.31 | 0.37 |
| Age of the youngest or only child, y (ref: ≤2) |  |  |  |  |
| 3–5 | 1.20 | 0.15 | 1.63* | 0.36 |
| 6–10 | 1.18 | 0.16 | 2.30** | 0.51 |
| 11–14 | 0.67* | 0.13 | 2.36** | 0.62 |
| >14 | 0.58* | 0.14 | 1.45 | 0.49 |
| Children with disorders or disabilities (ref: no) |  |  |  |  |
| Learning disabilities | 1.32 | 0.29 | 1.39 | 0.41 |
| Other disabilities | 2.07* | 0.75 | 5.85** | 2.16 |
| Chronic conditions | 0.40 | 0.29 | 1.46 | 0.91 |
| Autism spectrum disorders | 1.82 | 0.93 | 7.24** | 3.31 |
| Multiple conditions | 1.45 | 0.72 | 3.46* | 1.68 |
| Unspecified | 1.17 | 0.28 | 0.97 | 0.39 |
| How the parent sees her/his means after the pandemic (ref: better/unchanged) |  |  |  |  |
| More difficult | 1.10 | 0.11 | 1.01 | 0.16 |
| Much more difficult/seriously at risk | 1.27 | 0.25 | 1.54 | 0.42 |
| Running out of food more often (ref: no) | 1.23 | 0.36 | 1.45 | 0.53 |
| Children had more difficulty falling asleep (ref: no) | 1.22 | 0.15 | 1.51 | 0.32 |
| Children had more difficulty staying asleep (ref: no) | 1.38** | 0.15 | 1.24 | 0.20 |
| Children had more nightmares and sleep terrors (ref: no) | 1.12 | 0.14 | 1.86** | 0.32 |
| Changes in children’s food intake (ref: no) |  |  |  |  |
| More food | 1.05 | 0.12 | 1.35 | 0.22 |
| Less food | 1.48** | 0.20 | 1.25 | 0.28 |
| Children have missed going to school (ref: no) | 1.15 | 0.12 | 1.06 | 0.17 |
| Children have missed outdoor activities (ref: no) | 1.09 | 0.10 | · | · |
| Children have missed meeting friends (ref: no) | 1.15 | 0.15 | 1.12 | 0.22 |
| Children have missed playing sports (ref: no) | 1.16 | 0.12 | 1.11 | 0.17 |
| Any mood swing in your children? (ref: no) |  |  |  |  |
| Yes, more nervous, troubled, or sad | 1.56** | 0.23 | 2.77** | 0.80 |
| Yes, their mood has improved | 1.33 | 0.47 | 0.54 | 0.56 |
| Did your children have feelings of loneliness? (ref: no) |  |  |  |  |
| Yes, not putting it into words | 1.49** | 0.20 | 1.89** | 0.44 |
| Yes, putting it into words | 1.23 | 0.17 | 1.66* | 0.39 |
| *Constant* | 0.02** | 0.01 | 0.00** | 0.00 |

*Notes: Parents who answered “not sure” are excluded (n = 671); “·” denotes independent variables there were discarded in preliminary bivariate analysis (P>0.15).*

**Significant at the 5% level (P≤0.05); **Significant at the 1% level (P≤0.01).*

**Table A4. Percentage distribution of mood swings and information about distance learning, by child’s condition.**

| **Characteristic** | **No disorder**  **or disability**  **(*n* = 5485)** | **Learning**  **disabilities**  **(*n* = 257)** | **Other disabilities**  **(*n* = 90)** | **Chronic**  **conditions**  **(*n* = 70)** | **Autism**  **spectrum**  **(*n* = 49)** | **Multiple**  **conditions**  **(*n* = 49)** | **Unspecified**  **(*n* = 210)** |
| --- | --- | --- | --- | --- | --- | --- | --- |
| Any mood swing in your children? |  | (*P*<0.001)** | (*P*=0.551) | (*P*=0.023)* | (*P*=0.253) | (*P*=0.097) | (*P*=0.023)* |
| No | 25.4 | 14.0 | 21.1 | 37.1 | 16.3 | 12.2 | 18.1 |
| Yes, more nervous, troubled, or sad | 72.0 | 80.2 | 75.6 | 57.1 | 83.7 | 83.7 | 78.6 |
| Yes, their mood has improved | 2.5 | 5.8 | 3.3 | 5.7 | 0.0 | 4.1 | 2.9 |
| Unspecified | 0.1 | 0.0 | 0.0 | 0.0 | 0.0 | 0.0 | 0.5 |
| Feelings of inadequacy in supporting children’s distance learning ^(a)^ | (Ref.) | (*P*=0.005)** | (*P*=0.286) | (*P*=0.868) | (*P*=0.097) | (*P*=0.675) | (*P*<0.001)** |
| No | 43.9 | 32.8 | 34.8 | 47.3 | 35.9 | 40.9 | 26.7 |
| At times | 36.5 | 42.8 | 37.7 | 36.4 | 28.2 | 34.1 | 32.5 |
| Often | 19.3 | 24.4 | 27.5 | 16.4 | 35.9 | 25.0 | 13.1 |
| Unspecified | 0.3 | 0.0 | 0.0 | 0.0 | 0.0 | 0.0 | 27.7 |
| Feelings of inadequacy in helping children manage their anxiety related to distance learning ^(a)^ | (Ref.) | (*P*=0.046)* | (*P*=0.486) | (*P*=0.450) | (*P*=0.023)* | (*P*=1.000) | (*P*<0.001)** |
| No | 61.8 | 54.8 | 55.1 | 54.5 | 43.6 | 61.4 | 34.0 |
| Yes | 37.7 | 45.2 | 44.9 | 45.5 | 53.8 | 38.6 | 20.9 |
| Unspecified | 0.5 | 0.0 | 0.0 | 0.0 | 2.6 | 0.0 | 45.0 |
| Problems paying attention during distance-learning classes ^(a)^ | (Ref.) | (*P*<0.001)** | (*P*=0.841) | (*P*=0.567) | (*P*=0.098) | (*P*=0.106) | (*P*<0.001)** |
| No | 45.4 | 30.4 | 43.5 | 40.0 | 28.2 | 29.5 | 61.3 |
| Yes | 54.3 | 69.6 | 56.5 | 60.0 | 71.8 | 70.5 | 37.2 |
| Unspecified | 0.3 | 0.0 | 0.0 | 0.0 | 0.0 | 0.0 | 1.6 |
| In case of disorders or disabilities, has the child received specific support for distance learning? ^(a)^ | · | (Ref.) | (*P*=0.003)** | (*P*=0.109) | (*P*<0.001)** | (*P*=0.001)** | · |
| No | 0.0 | 75.2 | 55.1 | 80.0 | 35.9 | 47.7 | 0.0 |
| Yes | 0.0 | 24.0 | 43.5 | 16.4 | 56.4 | 52.3 | 0.0 |
| Unspecified | 0.0 | 0.8 | 1.4 | 3.6 | 7.7 | 0.0 | 0.0 |
| Not applicable | 100.0 | 0.0 | 0.0 | 0.0 | 0.0 | 0.0 | 100.0 |

*Legend. P-values from Fisher’s exact test indicate whether the distribution of a variable in one disease group is different from the distribution in the reference group (no disorders or disability).*

*^(a)^Families with no schoolchildren in the house are excluded (n = 2377).*

**Significant at the 5% level (P≤0.05); **Significant at the 1% level (P≤0.01).*
